# Impacts of Changing the Curriculum Design on the Examination Results of Anatomy and Physiology Course

**DOI:** 10.7759/cureus.24405

**Published:** 2022-04-23

**Authors:** Dur-e-Shewar Rehman, Ismail Memon, Nosheen Mahmood, Norah Alruwaili, Rasha Alhazzaa, Abdulmohsen Alkushi, Dunia Jawdat

**Affiliations:** 1 Anatomy, College of Science and Health Professions, King Saud Bin Abdulaziz University for Health Sciences, Riyadh, SAU; 2 Research, King Abdulaziz International Medical Research Center, Riyadh, SAU; 3 Pathology, College of Science and Health Professions, King Saud Bin Abdulaziz University for Health Sciences, Riyadh, SAU; 4 Physiology, College of Science and Health Professions, King Saud Bin Abdulaziz University for Health Sciences, Riyadh, SAU; 5 Immunology, College of Science and Health Professions, King Saud Bin Abdulaziz University for Health Sciences, Riyadh, SAU

**Keywords:** physiology, anatomy, comparison, modification, innovation, assessment

## Abstract

Introduction

Innovating strategies have become a compulsion in all fields associated with improved outcomes. Similarly, an innovation was introduced in the curriculum design and content to be tested for the Anatomy and Physiology course at the College of Science and Health Professions (COSHP), King Saud Bin Abdulaziz University for Health Sciences (KSAU-HS), in the spring semester of 2020. Before the COVID-19 pandemic, until the spring semester of 2019, two examinations were conducted as continuous assessments (Midterm I and II), followed by a comprehensive Final examination. In the spring semester of 2020, these examinations were replaced with Block I, II, and III examinations, respectively, with modified content and weightage. The Final examination was comprehensive and included 24 Anatomy, 21 Physiology lectures, and three case-based learning (CBL) sessions, whereas Block III included only eight Anatomy, seven Physiology lectures, and 1 CBL session. Midterm I and II weighed 20% each with a comprehensive examination of 35%, while Block I, II, and III were all 25% each. This study focuses on the impact of the curriculum modifications on the results of written examinations for preprofessional students enrolled at Riyadh, Jeddah, and Al-Ahsa campuses.

Methods

This retrospective study included data from 2356 male and female students from Riyadh, Jeddah, and Al-Ahsa. Data included Midterm I and II grades and Final examination grades for spring semester 2019 and Block I, II, and III examination grades for spring semester 2021. The results of the spring semester 2021 examinations were compared with the spring semester 2019 examination. The spring semester of 2020 was skipped to avoid the effect of online examinations during the COVID-19 restriction period. Data were analyzed using the statistical software SPSS version 23.0 (IBM Corporation, Armonk, NY, USA). Coefficient of variation (CV) compared spring semester 2019 and spring semester 2021 examination outcomes. The findings were analyzed concerning data related to gender, student groups, and campuses. An independent t-test of proportion was used to compare the CVs for spring 2019 and 2021.

Results

The overall comparison showed better results in the spring semester of 2021 (p-value < 0.01). Campus-wise, the results were significantly better for Riyadh (p-value < 0.01). The gender-wise study showed better performance from male students (p-value < 0.01). Concerning campus and gender, the results of male and female students of the Riyadh campus came out to be highly significant (p-value < 0.01).

Conclusions

Changing from Midterms to the Block system significantly improved the Block III examination results in spring semester 2021, particularly at the Riyadh campus. Overall, the changes remained helpful to all students. Further studies are needed to investigate the long-term effect of the curriculum changes.

## Introduction

Innovation is a new idea or amendment made in the current setup with the intention of showing improvements in the level of achievement. Innovation strategies are essential in every field [1]. Studies have been conducted considering innovations in Saudi universities, analyzing the quality of education, educational programs, teaching methods, applied research related to the industry, and universities’ financial sustainability, and developing partnerships and networks [2]. Since universities play a crucial role in the progress of society, they need to meet the criteria of modernization, improvement, and development [2]. Therefore, universities’ traditional academic structures need to be modified to cope with the challenges [3]. To promote innovation, a boost is required in the qualitative aspects of curriculum designing [2,4].

Students’ performance is considered a key indicator of the promising potential of a university [2]. Predicting students’ performance can help both the management and weak students work out personal, psychological, social, and other environmental factors and improve students’ performance to reduce the number of dropouts [5,6]. At the same time, mapping outcomes to particular topics in a university subject measuring student performance on assessments is vital [7]. Students’ academic performance/grades are known to be related to the assessment policy of the institution, and it is established that assessment drives learning [8-10].

We hypothesize that modifications in the curriculum design and assessment system are expected to improve the overall grades at the end of the semester.

## Materials and methods

This study was approved by King Abdulaziz International Medical Research Center (KAIMRC) (reference number IRBC/0693/21). The data included results of male and female Pre-Medicine (PMED), Pre-Dentistry (PDNT), Pre-Pharmacy (PPHR), and Pre-Applied Medical Sciences (PAMS) students registered in the Anatomy and Physiology course during the spring semester in 2019 and 2021 at College of Science and Health Professions (COSHP) Riyadh, Jeddah, and Al-Ahsa campuses. Those students who missed any of the examinations or continuous assessments were excluded.

This study was conducted to assess the examination results of students at the University Pre-Professional Program (UPPP) at the College of Science and Health Professions (COSHP), King Saud Bin Abdulaziz University for Health Sciences (KSAU-HS). KSAU-HS has three campuses in Riyadh, Jeddah, and Al-Ahsa. The first part of its medical sciences-related curriculum is offered in the UPPP, a two-year preparatory program consisting of four semesters [11]. In the academic year 2019-2020, curricular modifications were adopted. In the previous assessment, there were two Midterm written examinations (Midterm I and II) and a Final written examination in the form of multiple-choice questions (MCQs). The Final examination was comprehensive, covering all topics included in the Anatomy and Physiology course. In the spring semester of 2020, a Block system was introduced, and the course was divided into three blocks (Block I, II, and III). The content covered in a block was tested at the end of each block. This study analyzed the impact on semester examination results following the modifications in the curriculum and assessment for the Anatomy and Physiology course offered to PMED, PDNT, PPHR, and PAMS students.

In this comparative cohort study, a stratified sampling technique was used. It included the examination results of 2356 students from Riyadh, Jeddah, and Al-Ahsa. In spring 2019, there were 1302 enrollments, 679 male and 623 female students, while in spring 2021, there were 1054 enrollments, 548 male and 506 female students. Data included Midterm I and II and Final examination grades for spring semester 2019 and Block I, II, and III for spring semester 2021. The distribution of subjects is reported in Table 1.

Data collection

The results of Block I, II, and III written examinations of the spring semester of 2021 from all three campuses (Riyadh, Jeddah, and Al-Ahsa) were collected for PMED, PDNT, PPHR, and PAMS students. There were minor modifications made in the content of Midterm I versus Block I and Midterm II versus Block II. The major change was in the Final examination versus the Block III examination content to be tested, in which the Final examination was comprehensive, including all lectures of the course, while Block III excluded lectures included in Block I and II. These written examination results were compared with the Midterm I, Midterm II, and Final examination results of the spring semester of 2019. The spring semester of 2020 was skipped as all examinations were conducted online due to COVID-19.

Data management and analysis

Data were analyzed using the statistical software SPSS version 23.0 (IBM Corporation, Armonk, NY, USA). Coefficient of variation (CV) was reported to compare spring semester 2019 and spring semester 2021 examination outcomes of Midterm I versus Block I, Midterm II versus Block II, and Final examination versus Block III examination. These findings were analyzed in-depth concerning gender, student group, and campus (Tables 1-7).

An independent t-test of proportion was used to compare the CVs for spring 2019 and 2021. All p-values less than 0.05 were considered statistically significant to study the impact of the change in the curriculum and assessment.

## Results

In this study, to see the effects of modifications in the curriculum design and assessment, the male and female students’ results of Midterm I, Midterm II, and Final examinations conducted in the spring semester of 2019 are compared with the results of Block I, Block II, and Block III examinations conducted in the spring semester of 2021.

Table 1 depicts the gender, specialty group, and campus-wise details of students who appeared in the spring semesters 2019 and 2021 examinations. The number of male and female students in all groups (PMED, PDNT, PAMS, and PPHR) was higher in 2019 than in 2021. In both academic years, the number of male students was higher in PMED and PDNT groups, while more female students were in the PAMS group. For the PPHR group, male and female students’ numbers were nearly the same. The Al-Ahsa campus has only female PAMS students.

**Table 1:** Distribution of samples (n = 2356)

Semester	Gender	Student groups				Campus		
		PDNT	PMED	PAMS	PPHR	Riyadh	Jeddah	Al-Ahsa
Spring 2019 (n = 1302)	Male (n = 679)	73	338	212	56	507	172	0
	Female (n = 623)	58	202	307	56	378	189	56
Spring 2021 (n = 1054)	Male (n = 548)	30	299	192	27	360	188	0
	Female (n = 506)	31	190	255	30	255	179	72

Table 2 depicts the overall comparison of Midterm I, Midterm II, and Final examinations conducted in 2019, before the curricular changes, with Block I, II, and III examinations conducted in 2020, respectively, after curricular modifications. Generally, the students in spring semester 2021 (Block I, II, and III examinations) performed better than in spring semester 2019 (Midterm I and II and Final examinations). The Block III examination results in 2021 were significantly higher (p-value < 0.01) than the Final examination results in 2019.

**Table 2:** Comparison of spring semester 2019 and spring semester 2021 results

Assessments	CV (2019)	CV (2021)	p-value
Midterm I versus Block I	23	22	0.56
Midterm II versus Block II	27	25	0.27
Final versus Block III	31	23	<0.01*

Table 3 compares the examination results of PDNT, PMED, PAMS, and PPHR student groups who appeared in the spring semester 2019 examination (before the curricular changes) with those who appeared in the spring semester 2021 examination (after the curricular modifications). There was no significant difference in the students’ performance when each group was compared independently in all examinations before and after curriculum redesign.

**Table 3:** Comparison of spring semester 2019 and spring semester 2021 results for specialty/groups

Assessments	CV (2019)	CV (2021)	p-value
PDNT			
Midterm I versus Block I	24	20	0.53
Midterm II versus Block II	28	23	0.46
Final versus Block III	26	21	0.45
PMED			
Midterm I versus Block I	14	15	0.64
Midterm II versus Block II	20	19	0.68
Final versus Block III	21	20	0.69
PAMS			
Midterm I versus Block I	25	27	0.47
Midterm II versus Block II	28	29	0.73
Final versus Block III	28	25	0.29
PPHR			
Midterm I versus Block I	27	26	0.88
Midterm II versus Block II	31	24	0.34
Final versus Block III	32	25	0.34

The data in Table 4 compares the campus-wise effects of the curriculum and assessment changes on the students’ performance in the 2019 and 2021 examinations. The Riyadh campus students, all groups, showed significantly higher grades (p-value < 0.01) in the Block III examination in 2021 than in the Final examination in 2019. For Jeddah and Al-Ahsa campuses, the effects of curriculum modifications in the 2019 and 2021 examinations were insignificant for all three examinations.

**Table 4:** Comparison of spring semester 2019 and spring semester 2021 results for campuses

Assessments	CV (2019)	CV (2021)	p-value
Riyadh			
Midterm I versus Block I	25	25	0.99
Midterm II versus Block II	28	27	0.67
Final versus Block III	34	25	<0.01*
Jeddah			
Midterm I versus Block I	18	18	0.99
Midterm II versus Block II	19	22	0.33
Final versus Block III	22	20	0.52
Al-Ahsa			
Midterm I versus Block I	19	21	0.77
Midterm II versus Block II	27	20	0.35
Final versus Block III	20	22	0.78

Table 5 compares the curriculum modification effects on male and female students in all three examinations conducted in the spring semester of 2019 and 2021. Both male and female students (all groups) performed significantly better in the Block III examination (2021) as compared to the Final examination (2019) (p-value < 0.01). There was no significant difference between male and female students in Midterm I versus Block I and Midterm II versus Block II examinations in the 2019 and 2021 spring semesters.

**Table 5:** Comparison of spring semester 2019 and spring semester 2021 results for gender

Assessments	CV (2019)	CV (2021)	p-value
Male (overall)			
Midterm I versus Block I	26	25	0.68
Midterm II versus Block II	30	27	0.24
Final versus Block III	34	25	<0.01*
Female (overall)			
Midterm I versus Block I	20	19	0.67
Midterm II versus Block II	22	23	0.68
Final versus Block III	27	21	0.01*

The data in Table 6 compares the curriculum modification effects on male and female students in each PMED, PDNT, PAMS, and PPHR group in the 2019 and 2021 spring semester examinations. The overall comparison for each PMED male and female, PDNT male and female, PPHR male and female, and PAMS male and female came out to be insignificant for all three examinations for all campuses (Table 6).

**Table 6:** Comparison of spring semester 2019 and spring semester 2021 results for specialty and gender

Assessments	CV (2019)	CV (2021)	p-value
PMED (male)			
Midterm I versus Block I	14	17	0.29
Midterm II versus Block II	20	19	0.75
Final versus Block III	23	20	0.35
PMED (female)			
Midterm I versus Block I	13	13	0.99
Midterm II versus Block II	18	18	0.99
Final versus Block III	18	19	0.79
PDNT (male)			
Midterm I versus Block I	27	21	0.52
Midterm II versus Block II	31	24	0.47
Final versus Block III	27	18	0.33
PDNT (female)			
Midterm I versus Block I	17	18	0.90
Midterm II versus Block II	23	22	0.91
Final versus Block III	23	22	0.91
PAMS (male)			
Midterm I versus Block I	27	32	0.27
Midterm II versus Block II	32	32	0.99
Final versus Block III	31	28	0.50
PAMS (female)			
Midterm I versus Block I	21	21	0.99
Midterm II versus Block II	22	24	0.57
Final versus Block III	24	21	0.39
PPHR (male)			
Midterm I versus Block I	31	24	0.50
Midterm II versus Block II	32	20	0.25
Final versus Block III	37	24	0.23
PPHR (female)			
Midterm I versus Block I	20	27	0.45
Midterm II versus Block II	26	26	0.99
Final versus Block III	23	23	0.99

Table 7 shows the campus-wise effects of curriculum modification on male and female students in the spring semester 2019 and 2021 examination results. At the Riyadh campus, both male and female students’ results for the Block III examination (2021) versus the Final examination (2019) were significantly higher (p-value < 0.01). There was no significant difference between Midterm I versus Block I and Midterm II versus Block II examination results (Table 7). Independent results for all three examinations for male and female students in the Jeddah campus and female students in the Al-Ahsa campus were insignificant (Table 7).

**Table 7:** Comparison of spring semester 2019 and spring semester 2021 results for campus and gender

Assessments	CV (2019)	CV (2021)	p-value
Riyadh (male)			
Midterm I versus Block I	27	27	0.99
Midterm II versus Block II	31	28	0.34
Final versus Block III	36	27	<0.01*
Riyadh (female)			
Midterm I versus Block I	21	21	0.99
Midterm II versus Block II	23	25	0.56
Final versus Block III	30	21	<0.01*
Jeddah (male)			
Midterm I versus Block I	17	21	0.33
Midterm II versus Block II	20	24	0.36
Final versus Block III	23	20	0.48
Jeddah (female)			
Midterm I versus Block I	19	14	0.19
Midterm II versus Block II	18	20	0.62
Final versus Block III	21	19	0.63
Al-Ahsa (female)			
Midterm I versus Block I	19	21	0.77
Midterm II versus Block II	27	20	0.35
Final versus Block III	20	22	0.78

## Discussion

Both learners and facilitators aspire to enhance the educational experience and maximize its benefits. Hence, efforts were put forward at COSHP, KSAU-HS, for its Anatomy and Physiology courses offered to different groups of preprofessional students. An attempt was made to bring about innovation by revising the curriculum design. As a result, redistribution of the content to be tested in Block I, II, and III examinations saved students from being overwhelmed by covering the whole course as was done previously in the Final examination until the spring semester 2019.

In a pedagogical study, Marinović et al. (2009) [12] showed that the transition from longitudinal to Block system in the medical science courses improved the students’ grades, except in Anatomy, Physiology, and Pathology. In contrast, this study showed an improvement in students’ grades in the Anatomy and Physiology course in Block III spring semester 2021 examination compared to the spring semester 2019 examination. The difference between the results of Midterm I versus Block I and Midterm II versus Block II was insignificant as there were minor changes in Block I and II teaching material to be tested as compared to Midterm I and II (Table 2). The variability in the observations in different studies invites some critical work to be done in this area.

The groupwise comparisons of results of male and female students at all campuses for all examinations did not show a significant difference in the 2019 and 2021 examination results. The redistribution of students into groups reduced the sample size; the small sample size leads to insignificance [13]. Low CV values for each group in the spring semester of 2021 signify that the students benefitted from the change but statistically remained insignificant. The PPHR students gained maximum benefit compared to other groups (Table 3).

By modifying the curriculum and content assessed, this study showed that the college authorities succeeded in achieving the outcomes in terms of the grades of male and female students at the Riyadh campus. On the other hand, the Jeddah and Al-Ahsa campus students could not benefit from the curriculum changes. In fact, we do not know the exact reason behind this difference in Riyadh, Jeddah, and Al-Ahsa results. However, it has been observed previously that Jeddah and Al-Ahsa campuses have been showing better results compared to the Riyadh campus and were already performing better in the Final comprehensive examination. The comparison between Midterm I and II and Block I and II examinations was insignificant because there were minor changes in the content of Block I and II examination. Therefore, innovations in curriculum design need to be equally promoted and implemented on all campuses (Table 4).

Tables 5, 6, and 7 compare gender-wise effects as a whole, gender-wise differences in a particular group, and gender-wise differences on each campus, respectively, on examination results before and after innovations in curriculum design and assessment. In a study on fourth-grade medical students, Pavo et al. (2021) [14] concluded that gender difference seems not to affect final examination results and impact academic performance. Similarly, in this study, male and female students significantly performed better in the Final examination versus the Block III examination in the spring semester of 2021. Likewise, in the Midterm I and II and Block I and II examinations, the performance of the male and female students was not different (Table 5). The gender-wise comparison in a particular group did not indicate any significant difference because segregating the samples into groups decreased the sample size, and the small sample size led to insignificance [15,16].

Furthermore, in the UPPP at COSHP, the curriculum and the assessment system are unified at all three campuses. The same learning material is delivered to students, and the same examination papers are given to students. The examinations are conducted at the exact times. In this study, gender-wise comparison at each campus individually did not show any significant differences in 2019 and 2021 examination achievements in Jeddah and Al-Ahsa campuses, while a highly significant improvement was seen in male and female students of the Riyadh campus (Table 7).

## Conclusions

The curriculum design innovation significantly improved the Block III examination results in the spring semester of 2021, particularly at the Riyadh campus. Comparing the effect of curriculum innovation by segregating the students into specialty groups, male and female groups, and campus-wise groups did not significantly impact the examination performance. Overall, the change from Midterms to the Block system remained helpful to all students. We believe that these preliminary results show a promising improvement in the students’ performance. However, further studies investigating the long-term impact of curriculum modifications on larger student groups would make a more decisive effect.
